# Employing *Escherichia coli*-derived outer membrane vesicles as an antigen delivery platform elicits protective immunity against *Acinetobacter baumannii* infection

**DOI:** 10.1038/srep37242

**Published:** 2016-11-16

**Authors:** Weiwei Huang, Shijie Wang, Yufeng Yao, Ye Xia, Xu Yang, Kui Li, Pengyan Sun, Cunbao Liu, Wenjia Sun, Hongmei Bai, Xiaojie Chu, Yang Li, Yanbing Ma

**Affiliations:** 1 Laboratory of Molecular Immunology, Institute of Medical Biology, Chinese Academy of Medical Sciences & Peking Union Medical College, Kunming, 650118, China; 2 Yunnan Key Laboratory of Vaccine Research & Development on Severe Infectious Diseases, Kunming, 650118, China; 3 Yunnan Engineering Research Center of Vaccine Research and Development on Severe Infectious Diseases, Kunming, 650118 China

## Abstract

Outer membrane vesicles (OMVs) have proven to be highly immunogenic and induced an immune response against bacterial infection in human clinics and animal models. We sought to investigate whether engineered OMVs can be a feasible antigen-delivery platform for efficiently inducing specific antibody responses. In this study, Omp22 (an outer membrane protein of *A. baumannii*) was displayed on *E. coli* DH5*α*-derived OMVs (Omp22-OMVs) using recombinant gene technology. The morphological features of Omp22-OMVs were similar to those of wild-type OMVs (wtOMVs). Immunization with Omp22-OMVs induced high titers of Omp22-specific antibodies. In a murine sepsis model, Omp22-OMV immunization significantly protected mice from lethal challenge with a clinically isolated *A. baumannii* strain, which was evidenced by the increased survival rate of the mice, the reduced bacterial burdens in the lung, spleen, liver, kidney, and blood, and the suppressed serum levels of inflammatory cytokines. *In vitro* opsonophagocytosis assays showed that antiserum collected from Omp22-OMV-immunized mice had bactericidal activity against clinical isolates, which was partly specific antibody-dependent. These results strongly indicated that engineered OMVs could display a whole heterologous protein (~22 kDa) on the surface and effectively induce specific antibody responses, and thus OMVs have the potential to be a feasible vaccine platform.

Outer membrane vesicle (OMV) are closed spheroid vesicles with a diameter of 10–300 nm, generally released by Gram-negative bacteria. OMVs are produced by budding of the outer membrane; they contain mostly outer membrane molecules and enclose some periplasmic components. Many studies have revealed the potential of OMV-derived vaccines in inducing protective immunity against infections with pathogenic bacteria, such as *Neisseria meningitidis*, *Acinetobacter baumannii*, *Porphyromonas gingivalis*, *Salmonella enterica serovar Typhimurium*, *Helicobacter pylori*, and *Vibrio cholerae,* in mice^1^,^2^,^3^,^4^,^5^,^6^,^7^,^8^. Clinical application of OMV vaccines against *Neisseria meningitidis* serogroup B has been shown to have satisfactory safety and efficacy in countries such as the Netherlands^9^ and Norway^10^.

OMVs are naturally enriched with immunoactive components, including LPS, nucleotide acids, lipids, outer membrane proteins (OMPs), periplasmic proteins^11^, inner membrane proteins and cytoplasm proteins. Some of these components are pathogen-associated molecular patterns (PAMPs), which are presented to the innate arm or the first defensive line of the immune system and sensed by pattern recognition receptors (PRRs) such as Toll-like receptors (TLRs), thus driving the inflammatory response in conjunction with complement system activation^12^,^13^,^14^. It has been reported that the immunological characteristics of OMVs endow them with distinct capabilities to stimulate both innate and adaptive immunity *in vitro* and *in vivo*^1^,^3^,^4^,^7^,^8^. With the intrinsic advantages of a nanoscale delivery carrier, OMVs have the unique ability to enhance cellular uptake through ligand-dependent surface receptor cross-linking^15^. Thus, OMVs can effectively enhance immune response levels by promoting the uptake of antigens by antigen-presenting cells^16^.

OMVs have proven to be highly immunogenic and sufficiently potent to be an effective vaccine; however, their potential to be employed as a novel antigen delivery carrier have not been well identified. It was reported that heterologous proteins expressed in *E. coli* cells showing distribution in the periplasmic space were observed in the vesicle lumen of shedding OMVs^17^. In addition, expressed heterologous proteins that had the ability to anchor to the surface of *E. coli* cells were also presented on the derived OMVs^18^. Technologically, fusion with ClyA (a pore-forming hemolytic protein), HBP or AIDA can bring exogenous proteins to the surface of OMVs^19^,^20^,^21^,^22^,^23^. Moreover, exogenous proteins can also be presented in the inner lumen of OMVs by using an appropriate leader protein^17^. Thus, OMVs appeared to be highly tolerant of being modified by genetic manipulation and present exogenous proteins of interest.

In this study, the non-pathogenic *E. coli* DH5*α* strain was employed to prepare engineered OMVs displaying a previously identified immunogenic outer membrane protein of *A. baumannii,* Omp22^24^, which served as a representative antigen. Utilizing the intrinsic immunological and structural features of OMVs, we sought to investigate whether the engineered OMVs can present a functional heterologous protein, induce high titers of specific antibodies, and provide significant immune protection against lethal challenge with *A. baumannii* in a murine sepsis model, and thus to demonstrate the potential of using *E. coli* OMVs as a feasible antigen delivery platform.

## Results

### *E. coli* DH5*α* did not express the functional ClyA protein

**Figure 1:**
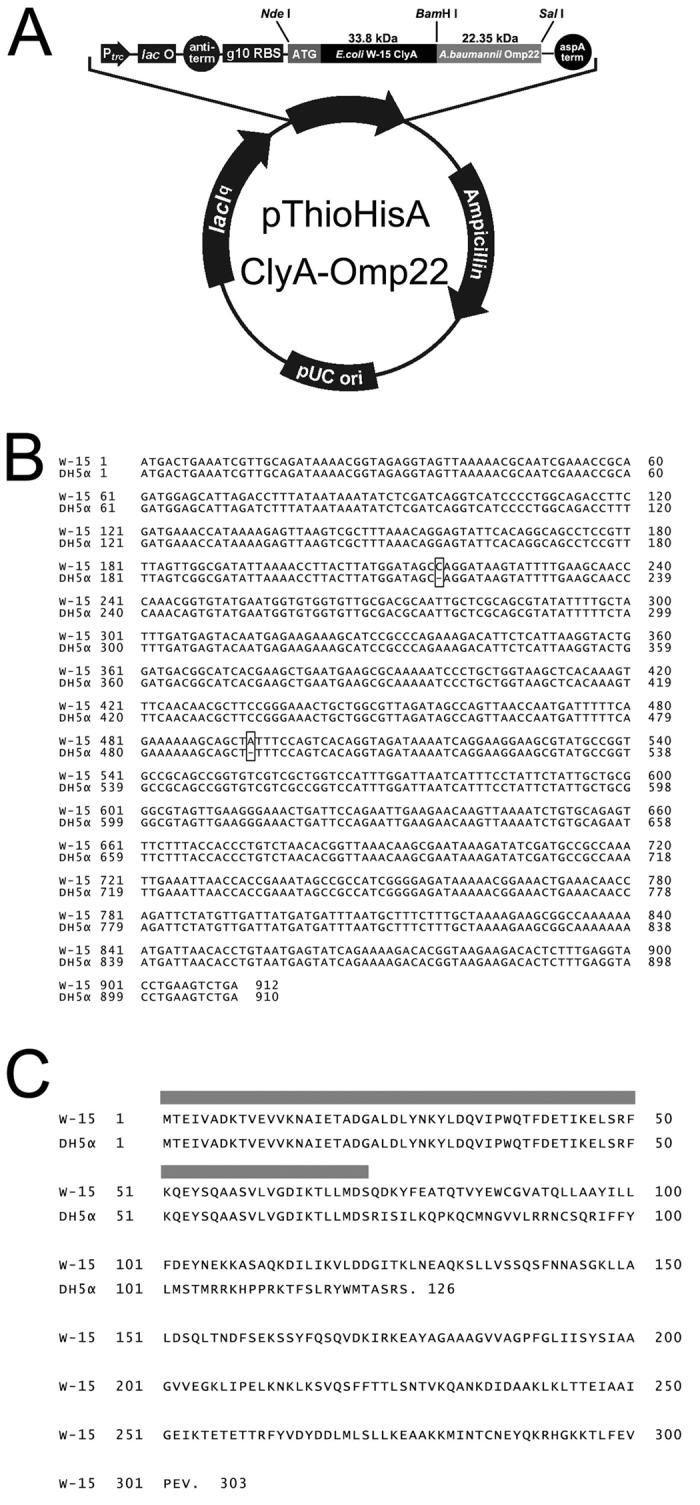
Genetic engineering of a ClyA-Omp22 fusion protein and nucleotide and amino acid sequences of *E. coli* DH5α- and W-15-derived ClyA. (**A**) Diagram of the recombinant plasmid expressing the fusion protein ClyA-Omp22. (**B**) Comparison of ClyA nucleotide sequences from the *E. coli* strains DH5α and W-15. The bracket indicates the location of missing bases in DH5α. (**C**) Comparison of the amino acid sequences of ClyA between the *E. coli* strains DH5α and W-15. Gray indicates identical amino acid sequences.

To obtain an *E. coli* ClyA gene, a clinical pathogenic W-15 strain was used as a PCR template in this study. The derived DNA sequence is identical to the reported *E. coli* ClyA gene sequences (Accession number: AF240780). In comparison, the ClyA gene from the *E. coli* DH5*α* strain lacked a C base at position 217 and an A base at position 493 (Fig. 1B and Supplementary Figure 1), resulting in a frame shift mutation and translation termination at the 126^th^ amino acid. The W-15 strain expressed the full-length and functional ClyA protein of 303 amino acids. A comparison between the amino acid sequences of ClyA between the two strains revealed only 72 identical amino acids at the N terminus (Fig. 1C), indicating *E. coli* DH5*α* lacks the functional ClyA.

### Exogenous Omp22 protein was successfully displayed on *E. coli* DH5α-derived OMVs

**Figure 2:**
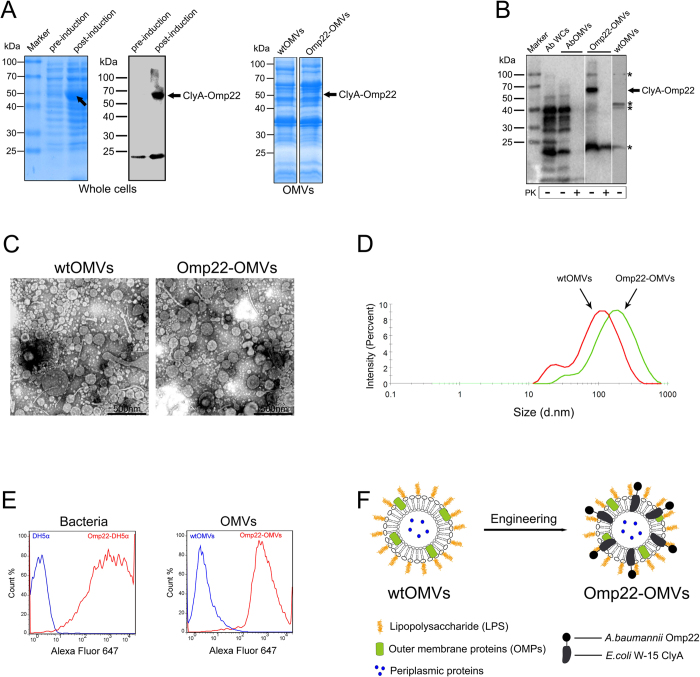
Presentation of Omp22 on the *E. coli*-derived OMVs. (**A**) SDS-PAGE and immunoblotting analyses of the expression of the ClyA-Omp22 fusion protein in *E. coli* DH5α within whole-cell protein samples (left), and SDS-PAGE shows its presence in OMVs (right). Arrows point to the protein bands representing ClyA-Omp22. (**B**) Immunoblotting analyses of displaying Omp22 on the surface of engineered OMVs using *A. baumannii* Ab1 OMV (*Ab*OMV) antiserum to provide detection antibodies. *Ab*OMVs, *E. coli* DH5α wild-type OMVs (wtOMVs), and recombinant Omp22-OMVs were treated with proteinase K (PK). The blots were imaged with the ChemiDoc™ MP imaging system (Bio-Rad). The figure is a representative result from three repeated experiments. Asterisks (*) indicate non-specific cross-reactive protein bands in *E. coli* wtOMVs. (**C**) Transmission electron microscope images of wtOMVs (left) and recombinant Omp22-OMVs (right). The bar indicates 500 nm. (**D**) Size distribution of OMVs according to diameter determined by dynamic light scattering. wtOMVs are in red and Omp22-OMVs are in green. (**E**) Immune fluorescence/flow cytometry analyses of the location of recombinant Omp22. DH5α cells and OMVs were surface stained with anti-Omp22 antibodies. Alexa Fluor 647-conjugated donkey anti-mouse IgG was used as the fluorescently labeled secondary antibody. (**F**) Schematic diagram of the construction of recombinant Omp22-OMVs.

As shown by SDS-PAGE, the whole cell (WC) sample of recombinant DH5α after induction with IPTG showed the appearance of an obvious band of approximately 56 kDa in comparison with a pre-induction sample. Immunoblotting showed that the post-induction sample of whole cell had a specific reaction band of approximately 56 kDa, comparing with the pre-induction sample, indicating the successful expression of the ClyA-Omp22 fusion protein (Fig. 2A, left). Further, the specific band in SDS-PAGE was digged out and analyzed by tandem mass spectrometry, and the result confirmed it was ClyA-Omp22 fusion protein. In addition, the engineered Omp22-OMVs showed a specific protein band of ClyA-Omp22 that was not present with wtOMVs (Fig. 2A right), which was also confirmed by immunoblotting (Fig. 2B, the fifth lane). Semi-quantitative analysis of the optical density of the protein bands revealed that the concentration of recombinant ClyA-Omp22 accounted for approximately 1% out of the total protein in the engineered OMVs.

To analyze whether the Omp22 protein was present on the surface or in the lumen, OMVs were digested with proteinase K (PK). Immunoblotting analyses using *Ab*OMV antiserum showed that, as a positive control, after surface proteins were hydrolyzed by PK digestion, *Ab*OMV samples lost reactivity with the *Ab*OMV antibody. The results indicated that only proteins exposed on the surface of OMVs elicited specific antibody production. For recombinant Omp22-OMVs, there was a specific reactive band present at approximately 56 kDa, which represented the ClyA-Omp22 fusion protein and was not observed in wtOMVs. After the Omp22-OMVs were treated with PK, the reactive band disappeared. In addition, four main protein bands appeared for the *E. coli* wtOMV sample, showing that some cross-reactions existed (asterisk) (Fig. 2B). Further, using flow cytometry to analyze DH5α cells and OMVs stained with anti-Omp22 antibodies, the results showed that only ClyA-Omp22-expressing cells and the derived OMVs presented strong staining (Fig. 2E). The results indicated that ClyA-Omp22 was likely to be displayed on the surface of the cells and the engineered OMVs.

Observation under an electron microscope showed that the morphology of wtOMVs and Omp22-OMVs was similar. This indicated that engineered Omp22-OMVs retained the structure of natural vesicles (Fig. 2C). DLS analysis showed that the maximum peak value was 124 nm and the polydispersity index (PdI) was 0.394. In contrast, the maximum peak value for Omp22-OMVs was 196.6 nm, and the PdI was 0.300 (Fig. 2D).

In a brief summary, using genetically engineering methods, the Omp22 protein was successfully presented through the leading of ClyA on DH5α-derived OMVs and most likely on the surface, which was diagramed in Fig. 2F.

### Specific antibody responses were induced efficiently in mice immunized with Omp22-OMVs

**Figure 3:**
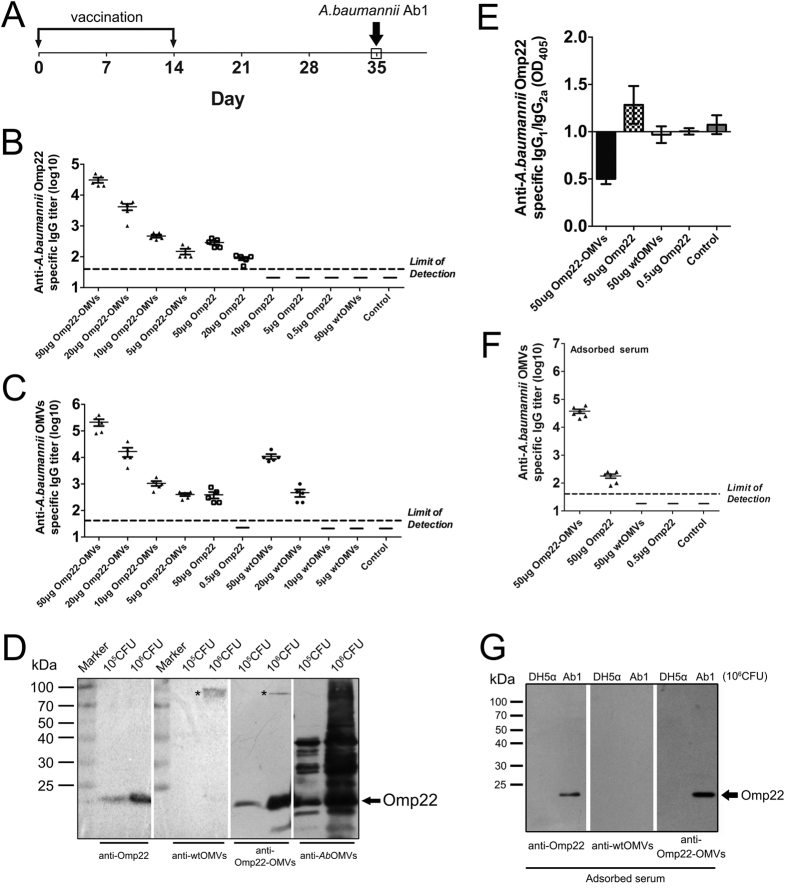
Induction of *A. baumannii* Omp22-specific antibodies by immunization with engineered Omp22-OMVs. (**A**) Immunization and bacterial infection procedures. The bracket indicates the time point of serum collection. (**B**) Detection of *A. baumannii* Omp22-specific antibody titers in the serum using ELISA. n = 5 mice/group. (**C**) Measurement of *A. baumannii* OMV-specific antibody titers in the serum using ELISAs. n = 5 mice/group. (**D**) Immunoblotting analysis. The samples were *A. baumannii* whole cells (10^5^ CFU or 10^6^ CFU). Different antisera were used to provide the primary antibodies. The blots were imaged with the ChemiDoc™ MP imaging system (Bio-Rad). The asterisk (*) indicates a cross-reactive protein band in *A. baumannii* Ab1 and *E. coli* DH5α. (**E**) The Omp22-specific ratio of IgG_1_/IgG_2a_ levels was measured using ELISAs. n = 5 mice/group. (**F**) Measurement of *A. baumannii* OMV-specific antibody titers in the adsorbed serum using ELISAs. n = 6 mice/group. (**G**) Immunoblotting analysis. The samples were whole cells (~10^6^ CFU) of *A. baumannii* or DH5α. Different adsorbed antisera were used to provide the primary antibodies.

Three weeks after the last immunization (day 35), blood samples were collected to detect the serum antibody titers (Fig. 3A). Using recombinant Omp22 to coat the microplates for ELISAs, the results showed that immunization with 50 μg of Omp22-OMVs (containing approximately 0.5 μg of Omp22 protein) produced an Omp22-specific antibody titer of approximately 30,000. Immunization twice with 50 μg of Omp22 protein produced only an antibody titer of approximately 300. In addition, 5 μg of Omp22-OMVs and 20 μg of Omp22 proteins could induce a low-level Omp22-specific antibody response. Additionally, immunization with 50 μg of wtOMVs or with 0.5 μg, 5 μg or 10 μg of recombinant Omp22 protein mixed with Alum, or immunization with Alum alone, did not produce detectable Omp22-specific antibody titers (Fig. 3B). Using *Ab*OMVs to coat the microplate, the ELISA results showed that immunization with 50 μg of Omp22-OMVs produced an anti-*Ab*OMV antibody titer of approximately 200,000. The titer of antiserum from mice immunized with 50 μg of Omp22 protein mixed with Alum was approximately 400. Notably, immunization with 50 μg of wtOMVs produced an antibody titer of approximately 10,000, which indicated that *E. coli* OMVs elicited antibody that cross-reacted with *A. baumannii* OMVs (Fig. 3C).

Both Omp22-OMV-immunized antiserum and recombinant Omp22-immunized antiserum specifically recognized the Omp22 protein in *A. baumannii* Ab1. Additionally, wtOMV-immunized antiserum could not recognize Omp22 in *A. baumannii* Ab1 but showed a weak immunological reaction with a protein with a molecular weight of approximately 90 kDa, which also cross-reacted with Omp22-OMV antiserum. As a positive control, *Ab*OMV antiserum strongly reacted with *A. baumannii* Ab1 proteins (Fig. 3D).

To evaluate the Th_1_/Th_2_ profiles responding to immunization with a vaccine employing OMVs as a carrier, Omp22-specific IgG_1_ and IgG_2a_ levels were determined using ELISA. The IgG_1_/IgG_2a_ ratio was calculated and showed that immunization with 50 μg of Omp22-OMVs elicited an IgG_2a_-biased response compared with immunization with 50 μg of recombinant Omp22 protein (Fig. 3E). The results implied that the OMV-based vaccine might facilitate eliciting Th_1_-like responses.

To exclude cross-reaction, the collected anti-sera were pre-adsorbed with DH5α cells. After the treatment, the *Ab*OMV-specific titer of antiserum from mice immunized with 50 μg of wtOMVs could not be detected in ELISA, while the Omp22-OMV antiserum still showed a high titer (Fig. 3F). Consistently, the non-specific cross-reactive bands could not be detected in immunoblotting, while the specific band for Omp22-OMVs was clearly present (Fig. 3G). These results clearly demonstrated that Omp22-OMV immunization induced Omp22-specific antibody responses in mice far more efficiently than did recombinant Omp22 proteins.

### Active immunization with Omp22-OMVs increased the survival rate of mice

**Figure 4:**
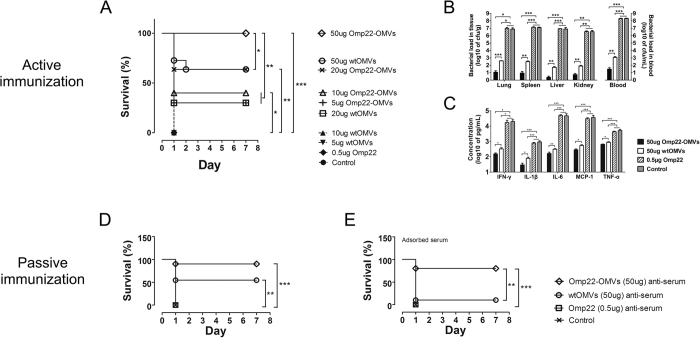
Immunization with engineered Omp22-OMVs increased the survival rates and reduced the bacterial loads and serum inflammatory cytokine levels. (**A**) The survival rates with different doses of Omp22-OMV or wtOMV immunization after *A. baumannii* Ab1 infection; n = 10 mice/group. (**B**) Bacterial load in the organs and blood; n = 5 mice/group. (**C**) Serum cytokine levels in mice; n = 6 mice/group. Serum from mice receiving Alum only was used as a control. (**D**) The survival rates with passive immunization with Omp22-OMV or wtOMV antisera after *A. baumannii* Ab1 infection; n = 10 mice/group. (**E**) The survival rates after administration of adsorbed antisera; n = 10 mice/group.

All the mice receiving the administration of an Alum adjuvant control, 0.5 μg of Omp22 mixed with Alum, 5 μg of wtOMVs or 10 μg of wtOMVs died within 24 hours after challenge with a clinical *A. baumannii* Ab1 isolate. The survival rates of the mice immunized with 50 μg, 20 μg, 10 μg or 5 μg of Omp22-OMVs (containing approximately 0.5 μg, 0.2 μg, 0,1 μg, or 0.05 μg of Omp22, respectively) were 100%, 63.6%, 40%, or 30%, respectively. Generally, the survival rates of the mice were positively correlated with the immunization doses of Omp22-OMVs. As a carrier control, immunization with 50 μg or 20 μg wtOMVs led to the survival of 63.6% or 35% of the mice, respectively, implying that *E. coli*-derived OMVs elicited cross-immunity to *A. baumannii* infection. The results showed that immunization with engineered Omp22-OMVs presenting Omp22 provided significant protection against lethal challenge with *A. baumannii* (Fig. 4A).

### Active immunization with Omp22-OMVs reduced the bacterial loads in major tissues

Mice immunized with 50 μg of Omp22-OMVs had significantly decreased bacterial loads in the blood in comparison with the mice immunized with 0.5 μg of Omp22 mixed with Alum or Alum alone. There was no significant difference found between the administration of 0.5 μg of Omp22 and Alum. Additionally, 50 μg of wtOMVs and Omp22-OMVs produced 1.7 × 10^5^-fold or 6.6 × 10^6^-fold reductions, respectively, compared to 0.5 μg of Omp22. The difference between Omp22-OMVs and wtMOVs was approximately 38-fold. Similarly, immunization with Omp22-OMVs reduced the bacterial loads in the lungs, spleen, liver, and kidneys by 7.8 × 10^5^-fold, 1.5 × 10^6^-fold, 3.6 × 10^6^-fold, and 6.2 × 10^5^-fold, respectively, compared to immunization with Omp22 and reduced the bacterial loads by 34-fold, 35-fold, 22-fold, and 14-fold compared to immunization with wtOMVs (Fig. 4B). These results indicated that immunization with engineered OMVs presenting Omp22 resulted in the effective clearance of *A. baumannii in vivo*.

### Active immunization with Omp22-OMVs reduced inflammatory cytokine levels in the serum

Consistent with the reduced bacterial loads, the levels of serum inflammatory cytokines in mice immunized with Omp22-OMVs were significantly lower than those in mice receiving Omp22 mixed with Alum or Alum alone. The serum concentrations of IFN-γ, IL-1β, IL-6, MCP-1, and TNF-α were 111-fold, 27-fold, 304-fold, 106-fold, and 6.2-fold lower, respectively, than those receiving Omp22. Furthermore, the serum concentrations of the cytokines were 2.1-fold, 2.6-fold, 1.9-fold, 1.9-fold, and 0.3-fold lower, respectively, than those receiving wtOMVs (Fig. 4C). These results suggested that immunization with Omp22-OMVs effectively reduced the production of inflammatory mediators stimulated by *A. baumannii* infection, probably due to the enhanced clearance of infecting bacteria.

### Passive immunization with Omp22-OMV antisera increased the survival rate of mice

Ninety percent of the mice immunized passively with Omp22-OMV antisera were protected from lethal *A. baumannii* Ab1 challenge. Additionally, the passive administration of wtOMV antisera led to the survival of 54.5% of the mice (Fig. 4D). This result strongly indicated that antisera elicited by *E. coli* OMVs significantly cross-reacted with *A. baumannii* and provided effective protection. To further clarify the effects of Omp22-specific antibodies, a parallel experiment employing pre-adsorbed antisera with DH5α cells was performed. The survival rate of the mice remained at 80% after passive immunization with adsorbed Omp22-OMV antisera, whereas only 10% of the mice survived after passive immunization with adsorbed wtOMV antisera (Fig. 4F). Furthermore, all mice receiving sera from Alum controls and 0.5 μg Omp22 administration died within 24 hours after challenge with clinical *A. baumannii* Ab1. These results showed that Omp22-OMVs elicited Omp22-specific antibodies that mediated significant protection against lethal challenge with *A. baumannii in vivo.*

### Omp22-OMV antiserum mediated the opsonophagocytosis of clinical *A. baumannii* isolates *in vitro*

**Figure 5:**
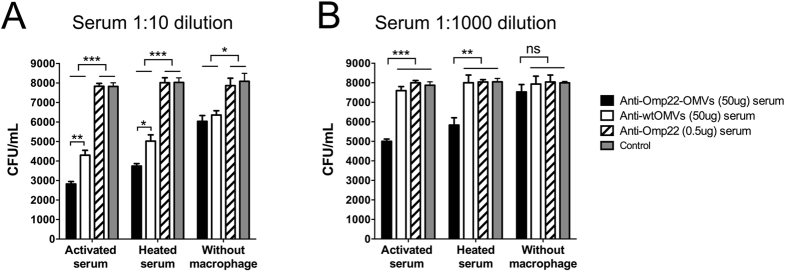
Opsonophagocytosis of clinical *A. baumannii* isolates mediated by Omp22-OMV antiserum *in vitro.* The possible bactericidal mechanism was analyzed using serum dilutions of 1:10 (**A**) and 1:1000 (**B**), with endogenous complement proteins inactivated or macrophage RWA264.7 cells removed. The results were expressed as the numbers of colony-forming units per ml of culture supernatant (CFU/ml). Experiments were performed in triplicate.

An *in vitro* opsonophagocytosis assay showed that at a 1:10 dilution of sera, both Omp22-OMV and wtOMV antisera mediated significant bactericidal activity against *A. baumannii* Ab1 compared to the serum derived from 0.5 μg of Omp22- or Alum control-immunized mice at serum dilutions of 1:10. The bacterial counts decreased by 64.1% for Omp22-OMV antiserum and 45.4% for wtOMV antiserum, respectively. However, there was a significant difference between Omp22-OMV and wtOMV antisera, indicating that the Omp22-specific antibody significantly contributed to bacterial killing. To further clarify the mechanism underlying the bactericidal activity mediated by the Omp22-OMV antisera, heat inactivation of sera and removal of macrophages from the reaction system were performed. Heat inactivation of endogenous complement reduced the bactericidal activity of Omp22-OMV antiserum from 64.1% to 53.4% and that of wtOMV antiserum from 45.4% to 37.5%. The removal of macrophages reduced the bactericidal activity of anti-Omp22-OMV antiserum and wtOMV antiserum to 25.3% and 21.4%, respectively (Fig. 5A). When the serum dilution was adjusted to 1:1000, Omp22-OMV antiserum still retained 36.5% killing activity compared to serum from mice immunized with the Alum control; in contrast, wtOMV antiserum showed only 3.5% killing activity (Fig. 5B). The results indicated that specific antibodies, complement proteins, and macrophage-dependent opsonophagocytosis might constitute an important bactericidal mechanism *in vivo.*

## Discussion

*A. baumannii* is a conditioned pathogen that is notorious for its easy acquisition of multi- or pan-drug resistance to clinically available antimicrobial agents. It was reported that subunit proteins of *A. baumannii,* including iron-regulated outer membrane proteins (IROMP)^25^, biofilm-associated protein (Bap)^26^, poly-N-acetyl-β-(1–6)-glucosamine (PNAG)^27^, trimeric autotransporter protein (Ata)^28^, outer membrane protein A (OmpA)^29^, and outer membrane protein W (OmpW)^30^, were potent vaccine candidates and provided effective immune protection against *A. baumannii* infection in animal models. We previously identified another antigenic outer membrane protein, Omp22, that could also provide immune protection against lethal challenge with clonally distinct clinical *A. baumannii* isolates in a murine sepsis model^24^. However, the immunogenicity of a subunit protein might be weak in humans when administered alone, and thus the protein must usually be combined with adjuvants^31^, conjugated to polysaccharide or vaccine carriers^32^,^33^, or conveyed by improved vaccine delivery systems^34^,^35^,^36^,^37^. Increasingly, success has come from innovations in the design of synthetic or biologically derived nanoparticle antigen carriers^37^,^38^,^39^ which allow for more efficient and targeted dissemination of the antigen to key immune cell populations^40^.

OMVs have received increased attention as an emerging and feasible vaccine carrier^41^ due to their distinct immunological and structural features, including nanometer-scale vesicle structure, self-adjuvant effectiveness, ability to be genetically modified, tolerance of presenting exogenous proteins and carrying immune stimulators^41^. It was reported that specific fusion of exogenous proteins to the C terminus of ClyA resulted in the display of functional proteins on the surface of OMVs, while fusion to the N terminus yielded unpredictable results^19^. Therefore, we fused Omp22 to the C terminus of ClyA to increase the chance of presenting a properly folded Omp22 protein. In this study, a domesticated laboratory *E. coli* strain DH5*α* was used to prepare the engineered OMVs for a proof-of-concept of employing OMVs as an effective antigen delivery carrier. The rationale for choosing DH5*α* is that, DH5*α* is a non-pathogenic host cell strain commonly adopted for expressing heterogeneous proteins using recombinant gene technology; further, DH5*α* has an incomplete ClyA gene which expresses a truncated protein of only the N-terminal 72 amino acids identical to ClyA, and we deduced that the absence of functional ClyA expression might facilitate the translocation of the recombinant protein ClyA-Omp22 to the outer membrane of *E. coli* cells and the presentation of interested antigen Omp22 on the derived OMVs. DSL analysis showed that the mean diameter of Omp22-OMVs was larger than that of wtOMVs, which may be attributed to the integration of ClyA-Omp22 in the outer membrane, which affects the formation of OMVs and increases the protein load on OMVs. However, electron microscopy demonstrated that the vesicular structure of Omp22-OMVs was similar to that of wtOMVs.

We used a method described by Kim *et al.* to determine the location of heterogeneous proteins on OMVs^19^. In their study, after incubation with PK, vesicle-associated fluorescence was abolished completely, suggesting that the functional GFP fused to ClyA was surface-exposed. And, they used immune electron microscopy to confirm the surface exposure of GFP. They further demonstrated that direct fusion of Bla, OPH, and anti-digoxin scFv to the C terminus of ClyA resulted in functional display of each protein on the surface of *E. coli* cells and their derived OMVs^19^. Some other groups also employed similar methods to fulfill the translocation and determine the location of interested proteins on OMVs. Kesty *et al.* used Tat to transport GFP to OMV lumen, and confirmed that GFP was therefore protected from degradation of pronase treatment^17^. Gujrati *et al.* sucessfully presented HER2 antibody on the surface of OMVs through using ClyA as leader protein, and the exposure of the antibody was demonstrated by ELISA with HER2 coated in the microplates^42^. Park *et al.* employed INP to transport multiple enzymes to the surface of OMVs, which was indicated by surface staining of the *E. coli* cells^43^. And, PspA was successfully transported to *Salmonella* OMVs lumen^44^ or surface^20^, and the location was determined by PK treatment. In a summary, the approach we used in this study that employing ClyA to facilitate the translocation of Omp22 to cells membrane and the presentation on the OMVs are proven to be successful by reported studies. And, the method of using proteinase (such as PK) treatment to show the location of the interested proteins on OMVs is broadly accepted and used. To provide more concrete data, we performed immune fluorescence staining and flow cytometry analysis and showed that ClyA-Omp22-expressing cells and the derived OMVs presented strong Omp22-specific staining, indicating it is most likely that Omp22 fused to ClyA was exposed on the surface of the engineered OMVs.

In this study, the engineered Omp22-OMVs contain only approximately 1% of Omp22 out of the total OMV proteins, which is similar to those reported by other groups (ranging from 0.32% to 0.8%)^17^,^44^. A much greater antibody response might be directed against commensal *E. coli* antigens. It is possibly a common shortcoming of bacterial vector vaccines as well as an OMVs vector vaccine described here. Some issues need to be further studied, including how long the antibodies against carrier and the immune memory will persist, whether pre-existing antibody will affect a repeated use of the vaccine, or how strong the antibody responses will produce significantly adverse influences. However, we have clearly demonstrated that OMVs-based vaccine efficiently induced specific protective antibody responses in the study. Worth to be mentioned, even if pre-existing anti-carrier antibodies have adverse influences on the repeated use of OMVs-based vaccines with different antigens, it is still optional and valuable to employ this vaccine platform in a prime-boost strategy of vaccination, as well as under the situation when specific immunity can not be induced effectively by conventional antigen such as a recombinant protein. We demonstrated that two doses of immunization with 50 μg Omp22-OMVs (containing only 0.1 μg Omp22) rapidly produced significantly higher Omp22-specific antibody responses than immunization with 50 μg recombinant Omp22 adjuvanted by Alum, while 0.5 μg recombinant Omp22 failed to elicit a visible response even if Alum was used. The results clearly demonstrated a prominent feature of OMVs that make them useful as vaccine carriers for inducing humoral immune responses far more efficiently than recombinant antigenic proteins. Considering that multiple proteins might be displayed simultaneously on OMVs through recombinant gene technology, and that low doses of antigen presented by OMVs might be sufficient to induce high titers of specific antibody, OMVs was showed the potential to be a convenient and powerful antigen delivery platform for a novel multivalent vaccine.

It was also shown in this study that immunization with *E. coli* wtOMVs did not produce Omp22-reactive antibodies but elicited antibodies that cross-reacted with *A. baumannii* proteins in ELISA and immunoblotting. After the treatment and adsorption with DH5α cells, the cross-reactivity was reduced to undetectable levels. Thus, the cross-reactivity was likely attributed to the presence of homologous proteins between *A. baumannii* and *E. coli,* such as the aforementioned 90 kDa protein. In a study with a sepsis model, active immunization with wtOMVs resulted in increased survival rates of mice as well as reduced bacterial load and inflammatory cytokine levels compared with the adjuvant controls. We speculated that the protective effects of *E. coli* wtOMV immunization against *A. baumannii* infection might be due to antibody cross-reactivity to homogeneous proteins and/or LPS, or due to activation of the innate immune response. To some extent, this affected our assessment of the contribution of the Omp22-specific antibody response to the clearance of *A. baumannii*. Thus, in a parallel experiment of passive antiserum immunization, sera pre-adsorbed with DH5α were used, by which antibodies against cross-reactive proteins and/or LPS were eliminated. The results showed a significant difference in the survival rate between pre-absorbed Omp22-OMV antiserum and wtOMV antiserum-administered mice (80% vs. 10%), strongly indicating that Omp22-OMV-elicited Omp22-specific immune responses contributed significantly to protection against *A. baumannii* infection. In addition, there were 4 bands on *E. coli* wtOMVs reacting with *Ab*OMVs antiserum in immunoblotting, which was an unexpected result from two not directly related bacterial strains. It indicated that some proteins in *A. baumanii* have similar antigen epitopes to those of *E. coli*, and thus *Ab*OMVs antiserum can recognize *E. coli* proteins in OMVs.

A key concern in all OMV vaccines is the presence of endotoxin. This study is designed mainly for a proof-of-concept, and is a first step towards development of a feasible new type of vaccine carrier. If necessary, endotoxin can be reduced or modified to less toxic variants to be applicable for use in man through genetically modifying host cells. The approach has been proven to work by the OMVs vaccine against *Neisseria meningitidis* serogroup B^45^,^46^,^47^,^48^ and the others^49^,^50^.

As shown by previous studies, specific antibody-mediated opsonophagocytosis or activation of the classical complement pathway may be common mechanisms of vaccination against *A. baumannii* infection with Omp22 or with other outer membrane proteins. Considering that *A. baumannii* is a conditioned pathogen and in general it does not invade host cells, cell-mediated immunity and blocking interactions between bacteria and host cells with neutralizing antibodies are not considered important in this case. An *in vitro* opsonophagocytosis assay confirmed that specific antibodies mediated the bactericidal effects of phagocytes such as macrophages; in particular, a significant difference was observed between Omp22-OMV antiserum and wtOMV antiserum (36.5% vs. 3.5%) when the serum was diluted 1:1000. Moreover, it was speculated that cross-reactive antibodies in wtOMV antiserum also mediated opsonophagocytosis, and thus, there was a significant difference from the control antiserum at a serum dilution of 1:10, but further dilution of the antisera to 1:1000 significantly reduced the effects caused by cross-reactive antibodies or nonspecific antibacterial components and made the effects of Omp22-specific antibody-mediated opsonophagocytosis dominant.

In summary, OMVs could display a complete Omp22 protein on their surfaces and maintain their proper conformation; immunization with Omp22-OMVs produced a strong Omp22-specific humoral immunity response in mice, even without the use of conventional adjuvants; and Omp22-OMV immunization protected mice from lethal challenge with a clinically isolated *A. baumannii* strain. The results indicated that OMVs could be utilized as a new and feasible antigen delivery platform for a vaccine to induce protective immunity.

## Materials and Methods

### Ethics statement

The animal experimental procedures were approved by the Ethics Committee of Animal Care and Welfare, Institute of Medical Biology, CAMS (Permit Number: SYXK (dian) 2010-0007) and were in accordance with the animal ethics guidelines of the Chinese National Health and Medical Research Council (NHMRC) and the Office of Laboratory Animal Management of Yunnan Province, China. All efforts were made to minimize animal suffering.

### Mice and bacterial strains

Female ICR mice (6–8 weeks of age) were raised and maintained at the Central Animal Care Services of the institute under specific pathogen-free (SPF) conditions. The *A. baumannii* ATCC 17978 strain was obtained from the American Type Culture Collection (ATCC). A clinical multi-drug resistant *A. baumannii* isolate, Ab1, was collected from the intensive care units (ICUs) of the Affiliated Hospital of Kunming Medical College (Kunming, China).

Nonpathogenic *E. coli* DH5*α* was purchased from Invitrogen, Inc., and pathogenic *E. coli* W-15 was also isolated from the local hospital (Kunming, China). The DNA fragment encoding ClyA was amplified by PCR and then sequenced. The primers were designed based on the published sequence of *E. coli* ClyA (Accession number: AF240780) and were as follows: ClyA-Forward (5′ CATATG ACT GAA ATC GTT GCA GAT A 3′) and ClyA-Reverse (5′ GGATCC GAC TTC AGG TAC CTC A 3′).

### Plasmid construction for expressing the ClyA/Omp22 fusion protein

Briefly, ClyA DNA from W-15 was ligated into the plasmid pThioHisA (Invitrogen) using the endonucleases *Nde*I and *BamH*I (Takara), and then the Omp22 gene (Accession number: CP000521; region: 1025531 to 1026184) amplified from *A. baumannii* strain ATCC 17978 was cloned between the *BamH*I and *Sal*I sites (Takara) (Fig. 1A). The primers Omp22-Forward (5′ GGATCC ATG CGT GCA TTA GTT AT 3′) and Omp22-Reverse (5′ GTCGAC TTA TTG TTT AGC ATA AAT GCT 3′) were used for amplification of the Omp22 gene.

### Preparation of engineered *E. coli* OMVs

OMVs were prepared according to the procedure described previously^6^. Briefly, *E. coli* cells were induced by isopropyl-β-d-thiogalactoside (IPTG, Sigma-Aldrich) at 30 °C for 20 h, and then the culture was centrifuged at 12,000 × g for 15 min. The supernatant was filtered through a 0.45-μm membrane (Millipore) and concentrated by ultra-filtration using a column with a 500,000 nominal molecular weight cutoff (500,000 NMWC, GE Healthcare). The concentrate was ultra-centrifuged at 200,000 × g for 2 h at 4 °C. The pellet containing OMVs was resuspended in phosphate-buffered saline (PBS; 0.02 mol/L phosphate buffer with 0.15 mol/L NaCl at pH 7.4) and then filtered through a 0. 45-μm membrane. OMVs were quantified using Bradford reagent (Thermo) according to the manufacturer’s instructions.

### Identification of engineered *E. coli* OMVs displaying Omp22

Expression of the ClyA-Omp22 fusion protein and its content in OMVs were analyzed by SDS-PAGE. Stained bands were analyzed by density scanning using Image lab software (Bio-Rad).

The expression of ClyA-Omp22 was detected by immunoblotting. Briefly, samples were separated by SDS-PAGE and then transferred to a PVDF membrane. Previously prepared *A. baumannii* Ab1 OMV (*Ab*OMV) antiserum was used as a primary antibody (diluted 1:1000)^6^, and horseradish peroxidase (HRP)-coupled anti-mouse IgG (Invitrogen) was used as a secondary antibody at a 1:10,000 dilution. The blots were developed with an ECL substrate (Thermo Scientific).

Tandem mass spectrometry MALDI-TOF-TOF analysis was used to identify the interested protein band in SDS-PAGE, which was performed by Kunming Biological Diversity Regional Center of Instruments, Chinese academy of sciences. The sample was digged out directly from the gel.

OMVs were measured by dynamic light scattering (DLS) using a Zetasizer Nano ZS (Malvern Instruments) for detecting the size distribution, which was reflected in the polydispersity index (PdI), ranging between 0.0 (monodispersed) and 1.0 (entirely heterodispersed)^51^,^52^. Next, the OMVs were observed and imaged using a transmission electron microscope (Hitachi) to analyze their morphology after fixing with 2.5% cold glutaraldehyde in 0.2 M sodium cacodylate buffer (pH 7.4) for 2 h at 4 °C and post-fixing with 1% osmium tetroxide in 0.1 M sodium cacodylate buffer (pH 7.4) for 1 h at 4 °C.

To demonstrate the distribution of Omp22, OMVs were treated with 0.1 μg/ml proteinase K (PK, Fermentas) for 1 h at 37 °C to degrade surface-exposed proteins^53^, and then the samples were analyzed by immunoblotting using *Ab*OMV antiserum as a primary antibody Further, immune fluorescence and flow cytometry was used to provide more concrete data. Briefly, bacteria and OMVs were surface stained with pre-adsorbed anti-Omp22-OMVs serum (1:1000 dilution). Alexa Fluor 647-conjugated donkey anti-mouse IgG (Millipore) was used as the fluorescently labeled secondary antibody (1:1,000 dilution). In each washing step, bacteria were collected directly by centrifuged at 10,000 × g for 5 min, and OMVs were collected using 50,000 MWCO ultrafiltration tube (Millipore) by centrifuged at 5,000 × g for 30 min. Flow cytometric analysis was performed using a BD LSRFortessa instrument and FlowJo software.

### Immunization and challenge of *A. baumannii* in a sepsis model

For active immunization, female ICR mice were immunized subcutaneously (s.c.) with 200 μl of Omp22-OMVs (5, 10, 20, and 50 μg) without adjuvant, wtOMVs (5, 10, 20, and 50 μg) without adjuvant, purified Omp22 protein (0.5, 5, 10, 20, and 50 μg) adjuvanted with 1 mg of Alum (Thermo scientific), or an Alum adjuvant control. The immunization was performed twice on days 0 and 14. On day 35, ~1 × 10^6^ CFU/200 μl of clinically isolated *A. baumannii* Ab1 cells with 10% porcine mucin (w/v; Sigma-Aldrich) were administered intraperitoneally (i.p.) to each mouse (Fig. 3A). For passive immunization, 100 μl of antisera collected from the mice immunized with 50 μg of Omp22-OMVs was injected intravenously (i.v.) into the mice 1 hour before challenge with *A. baumannii* Ab1; the sera collected from the mice receiving 50 μg of wtOMVs, 0.5 μg of Omp22 and adjuvant only served as the controls. Next to exclude a nonspecific cross-reaction, a parallel experiment was performed using pre-adsorbed antisera. Briefly, the antisera were incubated with 10^10^ CFU/ml DH5α cells for 3 h at 4 °C, followed by centrifugation at 12,000 × g for 10 min at 4 °C, and then filtered through a 0.45 μm membrane.

### Antibody responses and cytokine levels

The serum samples were collected on day 35. Omp22 or *Ab*OMV-specific IgG responses were measured by enzyme-linked immunosorbent assays (ELISA). Briefly, 96-well plates were coated overnight with 25 μg/100 μl recombinant *A. baumannii* Omp22 protein or *Ab*OMVs per well. The plates were incubated with the collected serum samples or pre-absorbed serum and then incubated with anti-mouse IgG secondary antibodies (diluted at 1:10,000, Santa Cruz) and developed with alkaline phosphatase substrate. The titer was defined as the highest dilution at which the optical density at 405 nm was at least 0.1 above that of the background wells in which serum samples were replaced with PBS. The Omp22-specific IgG_1_/IgG_2a_ ratio in the serum was also measured. Briefly, after sample incubation, either biotinylated goat anti-mouse IgG_1_ or IgG_2a_ (Life Technologies) was used as a secondary antibody, and the reaction was developed using an avidin–phosphatase/substrate system.

Next, immunoblotting assays were performed to check the reactive specificity of the antisera. The WC sample of Ab1 was separated by SDS-PAGE, transferred to a membrane and incubated with the previously prepared *Ab*OMV antisera (at a dilution of 1:1000)^6^ or the antisera collected from mice immunized with 50 μg Omp22, wtOMVs, or Omp22-OMVs. Horseradish peroxidase (HRP)-coupled anti-mouse IgG (Invitrogen) was used as the secondary antibody, and the blots were developed with an ECL substrate (Thermo Scientific). To avoid cross-reactivity, pre-adsorbed antisera were used in a parallel assay with DH5α, and Ab1 WCs were used as samples.

The concentrations of IFNγ, IL-1β, IL-6, MCP-1, and TNFα in the serum were detected using ELISA. Paired capture and biotinylated detection antibodies purchased from eBioscience, Inc. (USA) were used according to the supplier’s instructions.

### Survival rate and bacterial burden

The mice were monitored continuously for seven days to determine the survival rate after challenge with Ab1. The bacterial burdens in the blood and major organs (lung, spleen, liver, and kidney) of the mice were measured 12 h after challenging with *A. baumannii* by plating 10-fold dilutions on LB plates. The plates were incubated at 37 °C overnight, and the CFUs were counted. The results are expressed as CFU/g tissue or CFU/ml blood.

### Opsonophagocytic assays

The experiment was performed according to a procedure reported previously^6^. Briefly, activated murine macrophage RAW264.7 cells (~1.5 × 10^5^ cells/well), the *A. baumannii* isolate Ab1, (~8 × 10^3^/well), and serum samples (10 μl/well) were added to microwells and incubated for 1 h with gentle shaking. Finally, the mixtures were diluted and plated for CFU counting. In this assay, the pooled sera obtained from mice immunized with 0.5 μg of Omp22, 50 μg of Omp22-OMVs, 50 μg of wtOMVs or an adjuvant control were diluted to final concentrations of 1:10 and 1:1000. The sera were subjected to heat treatment at 56 °C for 30 min to inactivate the endogenous complement components.

### Statistical analyses

All statistical analyses were performed using GraphPad Prism 6.0 (GraphPad Software, Inc.). The survival rates were compared using the non-parametric log-rank test. One-way ANOVA with Tukey’s multiple comparison test was used to analyze the bacterial burden, cytokine levels and opsonic activity. Differences were considered significant if the *P* value was < 0.05 (*P < 0.05; **P < 0.01; and ***P < 0.001). All graphed values represent the mean, and the error bars represent standard error.

## Additional Information

**How to cite this article**: Huang, W. *et al.* Employing *Escherichia coli*-derived outer membrane vesicles as an antigen delivery platform elicits protective immunity against *Acinetobacter baumannii* infection. *Sci. Rep.* **6**, 37242; doi: 10.1038/srep37242 (2016).

**Publisher’s note:** Springer Nature remains neutral with regard to jurisdictional claims in published maps and institutional affiliations.

## Supplementary Material

Supplementary Information
