# Supplementary material for: Employing Escherichia coli-derived outer membrane vesicles as an antigen delivery platform elicits protective immunity against Acinetobacter baumannii infection
Source: Sci Rep. 2016 Nov 16;6:37242. doi: 10.1038/srep37242 (PMC5110958; doi:10.1038/srep37242)

Employing *Escherichia coli*-derived outer membrane vesicles as an antigen delivery platform elicits protective immunity against *Acinetobacter baumannii* infection

Weiwei Huang[1,2,3], Shijie Wang[1,2,3], Yufeng Yao[1,2,3], Ye Xia[1,2,3], Xu Yang[1,2,3], Kui Li[1,2,3], Pengyan Sun[1,2,3], Cunbao Liu[1,2,3], Wenjia Sun[1,2,3], Hongmei Bai[1,2,3], Xiaojie Chu[1,2,3], Yang Li[1,2,3], Yanbing Ma[1,2,3*]

[1] Laboratory of Molecular Immunology, Institute of Medical Biology, Chinese Academy of Medical Sciences & Peking Union Medical College; Kunming, China 650118, [2] Yunnan Key Laboratory of Vaccine Research & Development on Severe Infectious Diseases; Kunming, China 650118, [3] Yunnan Engineering Research Center of Vaccine Research and Development on Severe Infectious Diseases, Kunming, China 650118.

***Corresponding:**

Prof. Yanbing Ma

Laboratory of Molecular Immunology,

Institute of Medical Biology,

Chinese Academy of Medical Sciences & Peking Union Medical College,

Kunming, China 650118

Phone: +86 871 68339287

Fax: +86 871 68334483

Email: yanbingma1969@126.com

Supplementary figure legends

Supplementary figure 1. The DNA sequencing graph of ClyA gene

DNA sequencing was used to analyze the ClyA gene from a clinical pathogenic *E.coli* W-15 and non-pathogenic *E.coli* DH5a, the result shows the ClyA gene from the DH5*α* strain lacked a C base at position 217 and an A base at position 493 (Black arrow).

Supplementary Figure 1

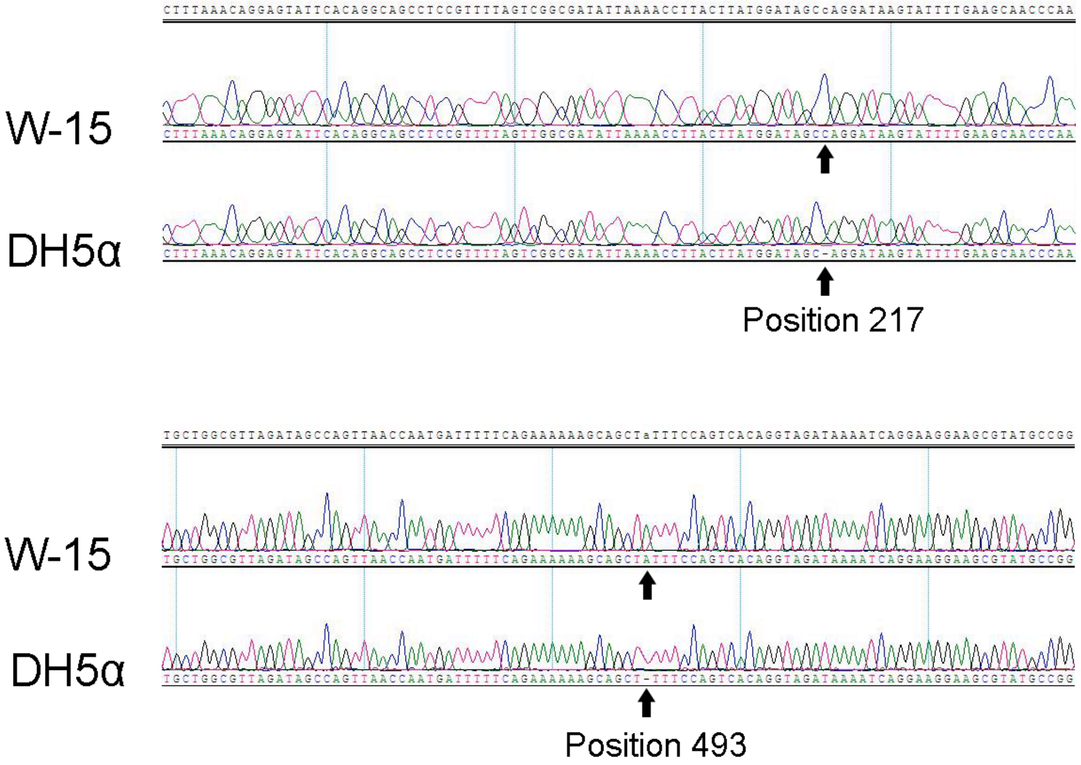

Supplement: Supplementary Information [file srep37242-s1.pdf]
